# Hepatoprotective Limonoids from Andiroba (*Carapa guianensis*)

**DOI:** 10.3390/ijms17040591

**Published:** 2016-04-19

**Authors:** Kiyofumi Ninomiya, Seiya Miyazawa, Kaiten Ozeki, Natsuko Matsuo, Osamu Muraoka, Takashi Kikuchi, Takeshi Yamada, Reiko Tanaka, Toshio Morikawa

**Affiliations:** 1 Pharmaceutical Research and Technology Institute, Kindai University, 3-4-1 Kowakae, Higashi-osaka, Osaka 577-8502, Japan; ninomiya@phar.kindai.ac.jp (K.N.); t0m0n1.aym-iv0.akrmn@ezweb.ne.jp (S.M.); kaiten.ozeki@gmail.com (K.O.); 1111610153n@kindai.ac.jp (N.M.); 2 Antiaging Center, Kindai University, 3-4-1 Kowakae, Higashi-osaka, Osaka 577-8502, Japan; 3 Laboratory of Pharmaceutical Organic Chemistry, Faculty of Pharmacy, Kindai University, 3-4-1 Kowakae, Higashi-osaka, Osaka 577-8502, Japan; muraoka@phar.kindai.ac.jp; 4 Laboratory of Medicinal Chemistry, Osaka University of Pharmaceutical Sciences, 4-20-1 Nasahara, Takatsuki, Osaka 569-1094, Japan; t.kikuchi@gly.oups.ac.jp (T.K.); yamada@gly.oups.ac.jp (T.Y.)

**Keywords:** hepatoprotective effect, limonoid, andiroba, *Carapa guianensis*, Meliaceae, structural requirement

## Abstract

Three gedunin-type limonoids, gedunin (**1**), 6α-acetoxygedunin (**2**), and 7-deacetoxy-7-oxogedunin (**3**), which were isolated from the seed and flower oils of andiroba (*Carapa guianensis* Aublet, Meliaceae), exhibited hepatoprotective effects at doses of 25 mg/kg, p.o. against d-galactosamine (d-GalN)/lipopolysaccharide (LPS)-induced liver injury in mice. To characterize the mechanisms of action of **1**–**3** and clarify the structural requirements for their hepatoprotective effects, 17 related limonoids (**1**–**17**) isolated from the seed and/or flower oils of *C. guianensis* were examined in *in vitro* studies assessing their effects on (i) d-GalN-induced cytotoxicity in primary cultured mouse hepatocytes, (ii) LPS-induced nitric oxide (NO) production in mouse peritoneal macrophages, and (iii) tumor necrosis factor-α (TNF-α)-induced cytotoxicity in L929 cells. The mechanisms of action of **1**–**3** are likely to involve the inhibition of LPS-induced macrophage activation and reduced sensitivity of hepatocytes to TNF-α; however, these compounds did not decrease the cytotoxicity caused by d-GalN. In addition, the structural requirements of limonoids (**1**–**17**) for inhibition of LPS-induced NO production in mouse peritoneal macrophages and TNF-α-induced cytotoxicity in L929 cells were evaluated.

## 1. Introduction

**Figure 1:**
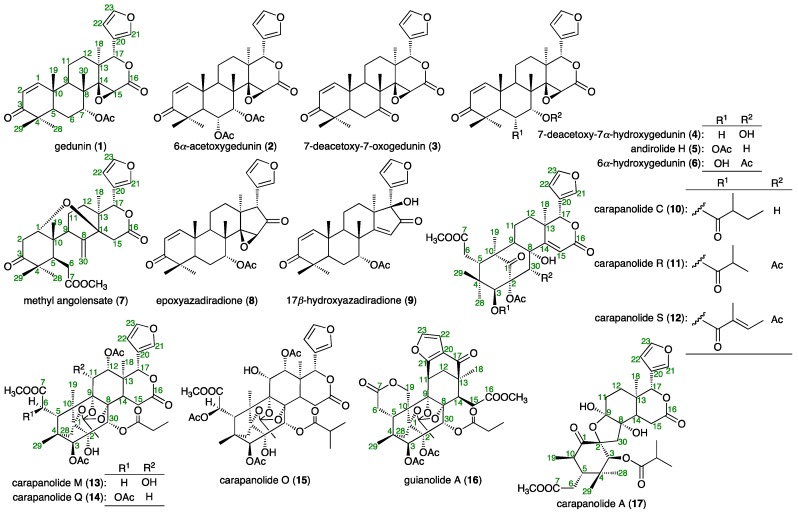
Limonoids (**1**–**17**) from seed and flower oils of *C. guianensis*.

The Meliaceae family is recognized as a rich source of limonoids, which have attracted attention from biogenetic and synthetic perspectives [1,2,3]. *Carapa guianensis* Aublet (Meliaceae), known locally as andiroba and Brazilian mahogany, is distributed in the tropical rainforests of countries such as Brazil and Colombia, *etc*. The woody four-cornered andiroba nut has four cells, each of which contains two to three seeds with oil-rich kernels. Extracts of the bark, flowers, and seeds have been used for centuries by the Amazonian people and exhibit various effects: anti-bacterial [4], anti-cancerous [5], anti-tumor [6], anti-fungal [6], insect repellent [7], analgesic [8], anti-malarial [9], anti-inflammatory [10], antiallergic [11], and anti-plasmoidal effects [12], as well as acute and subacute toxicity [13]. In the course of our studies on the chemical constituents from *C. guianensis* (*Carapa guianensis*) [14,15,16,17,18,19,20,21,22,23], we have isolated several limonoids, including andirolides A–Y from the flower oil [14,15,16,17], carapanolides A–X [18,19,20,21,22], and guianolides A and B [23] from the seed oil. We have also reported that several limonoids from *C. guianensis* showed cytotoxic [14,16,18,19,23], antimalarial [15], anti-inflammatory [17,20,22], and triglyceride metabolism-promoting activities [21]. We further evaluated the principal gedunin-type limonoids from the flower oil of *C. guianensis*, gedunin (**1**) [15], 6α-acetoxygedunin (**2**) [14], and 7-deacetoxy-7-oxogedunin (**3**) [14], which were found to have protective effects against liver injury induced by d-galactosamine (d-GalN)/lipopolysaccharide (LPS) in mice. To characterize the mechanisms of action of limonoids and the structural requirements for their hepatoprotective effects, 17 related limonoids isolated from the flower oil of *C. guianensis*, such as 7-deacetoxy-7α-hydroxygedunin (**4**) [21], andirolide H (**5**) [15], 6α-hydroxygedunin (**6**) [15], and methyl angolensate (**7**) [15], as well as limonoids isolated from the seed oil including epoxyazadiradione (**8**), 17β-hydroxyazadiradione (**9**) [21], carapanolides C (**10**) [19], R (**11**) [21], S (**12**) [21], M (**13**) [21], Q (**14**) [21], and O (**15**) [21], guianolide A (**16**) [23], and carapanolide A (**17**) [18] (Figure 1). This study reports the hepatoprotective effects and possible mechanisms of action of **1**–**3**, as well as the structural requirements for their hepatoprotective effects.

## 2. Results and Discussion

### 2.1. Isolation

In previous studies, compounds **1**–**7** were isolated from the flower oil of *C. guianensis* [14,15,16,17], whereas compounds **8**–**17** were obtained from the seed oil [18,19,20,21,22]. In the present study, principal limonoids (**1**–**3**) were identified from the seed oil using normal phase silica gel column chromatography followed by HPLC or recrystallization.

### 2.2. Protective Effects of Principal Limonoids (**1**, **2**, and **3**) on Liver Injury Induced by d-GalN/LPS in Mice

d-GalN/LPS-induced liver injuries are known to develop through immunological responses [24] that progress via two steps. First, expression of inhibitors of apoptosis proteins (IAPs) is inhibited by administration of d-GalN through depletion of uridine triphosphate and increased sensitivity of hepatocytes to tumor necrosis factor-α (TNF-α. Second, release of pro-inflammatory mediators [nitric oxide (NO) and TNF-α from LPS-activated macrophages (Kupffer’s cells) occurs. Apoptosis of hepatocytes induced by TNF-α plays an important role in d-GalN/LPS-induced liver injury [25]. In our previous investigation of compounds from natural medicines possessing hepatoprotective activity, we reported that sesquiterpenes and diarylheptanoids from *Curcuma zedoaria* [26,27,28,29], saponins from *Panax notoginseng* [30], coumarins from *Angelica furcijuga* [31], acid amides from *Piper chaba* [32,33,34], acylated phenylethanoids from *Cistanche tubulosa* [35], and triterpenes from *Potentilla anserina* [36] exhibited significant protective effects against liver injuries induced by d-GalN/LPS in mice.

First, the *in vivo* hepatoprotective effects of the principal limonoid constituents, gedunin (**1**), 6*α*-acetoxygedunin (**2**), and 7-deacetoxy-7-oxogedunin (**3**), were evaluated. As shown in Table 1, **1**–**3** at a dose of 25 mg/kg, p.o. clearly prevented mortalities and significantly inhibited the increase in serum levels of aspartate aminotransaminase (sAST) and alanine transaminase (sALT), which served as markers of acute liver injury [37,38,39]. Notably, **1**–**3** were more potent than positive compounds curcumin [36,40,41,42] and silybin [43,44], which are well recognized as naturally-occurring hepatoprotective products.

### 2.3. Effects on d-GalN-induced Cytotoxicity in Primary Cultured Mouse Hepatocytes

As a part of our studies to characterize the hepatoprotective compounds from natural medicines, we have investigated several constituents showed inhibitory effect on d-GalN-induced cytotoxicity in primary cultured hepatocytes [26,27,29,31,32,33,34,35,36,43,44,45,46,47,48,49,50,51,52,53,54,55]. Since the principal limonoid constituents from the flower oil of *C. guianensis* (**1**–**3**) showed hepatoprotective effects against d-GalN/LPS-induced liver injury in mice (*vide ante*), the inhibitory effect of limonoids (**1**–**17**) on d-GalN-induced cytotoxicity in primary cultured mouse hepatocytes were examined using the 3-(4,5-dimethylthiazol-2-yl)-2,5-diphenyltetrazolium bromide (MTT) assay. As shown in Table 2, these limonoids (**1**–**17**) and curcumin [26,27,29] did not reduce d-GalN-induced cytotoxicity in primary mouse hepatocytes at concentrations of up to 100 μM, whereas silybin (IC_50_ = 38.8 μM) significantly inhibited cytotoxicity [33,35,36]. Thus, these limonoids (**1**–**17**) did not affect cytotoxicity caused by d-GalN.

### 2.4. Effects on LPS-induced NO Production in Mouse Peritoneal Macrophages

The effects of limonoids (**1**–**17**) on NO production were examined to provide an estimation of macrophage activation levels in LPS-treated mouse peritoneal macrophages. As shown in Table 3, gedunin-type, gedunin (**1**, IC_50_ = 4.6 µM) [17], 6*α*-acetoxygedunin (**2**, 7.9 µM) [17], 7-deacetoxy-7-oxogedunin (**3**, 12.8 µM) [17], 7-deacetoxy-7*α*-hydroxygedunin (**4**, 8.7 µM) [17], andirolide H (**5**, 9.4 µM), 6*α*-hydroxygedunin (**6**, 19.1 µM) [17], epoxyazadiradione (**8**, 8.2 µM), 17*β*-hydroxyazadiradione (**9**, 20.3 µM), mexicanolide-type, carapanolides R (**11**, 68.3 µM) and S (**12**, 15.5 µM), phragmalin-type limonoids, carapanolides M (**13**, 41.6 µM), Q (**14**, 38.0 µM), and O (**15**, 46.0 µM), and guianolide A (**16**, 77.9 µM) significantly inhibited NO production without notable cytotoxic effects at the effective concentration. The NO production inhibitory activities of gedunin-type limonoids (**1**–**6**, **8**, and **9**, IC_50_ = 4.6–20.3 µM) were higher than those of other skeletal-type limonoids such as andirobin-type (**7**, > 100 µM), mexicanolide or 9,10-*seco*-mexicanolide-type (**10**–**12** and **17**, 15.5 – >100 µM), and phragmalin-type limonoids (**13**–**16**, 38.0–77.9 µM). The potencies of **1**–**6**, **8**, and **12** were higher than that of the NO synthase inhibitor, *N*^G^-monomethyl-l-arginine (l-NMMA, IC_50_ = 36.0 µM) and equivalent to that of caffeic acid phenethyl ester (CAPE, 11.0 µM), an inhibitor of nuclear factor-κB activation.

The structural requirements of gedunin-type limonoids were assessed: (i) 6α-acetoxy and 6α-hydroxy moieties reduced the activity [gedunin (**1**) > 6α-acetoxygedunin (**2**), 6α-hydroxygedunin (**6**)]; (ii) compounds with 7α-acetoxy group exhibited higher activity than those with 7α-hydroxy or 7-keto groups [**1** > 7-deacetoxy-7-oxogedunin (**3**), 7-deacetoxygedunin (**4**)]; (iii) compounds with an α,β-epoxy-δ-lactone moiety in the D-ring exhibited higher activity than those with an α,β-epoxy or α,β-unsaturated cyclopropane moieties [**1** > epoxyanadiradione (**8**), 17β-hydroxyazadiradione (**9**)]. For mexicanolide- and phragmalin-type limonoids, the following relationships were suggested: the 30-*O*-acyl moieties were essential for the activity [carapanolide C (**10**) ≪ carapanolides R (**11**), S (**12**), M (**13**), Q (**14**), and O (**15**)], whereas a 6-acetoxy moiety did not affect the activity [**13** ≒ **15**], while an 11*α*-hydroxy moiety reduced the activity [**14** > **15**].

### 2.5. Effects on TNF-α-induced Cytotoxicity in L929 Cells

The effects of the limonoids (**1**–**17**) on the sensitivity of hepatocytes to TNF-α were assessed by measuring TNF-α-induced decreases in the viability of L929 cells, a TNF-α-sensitive cell line [56], by using the MTT assay. In the absence of a test sample, the cells incubated with 1 ng/mL TNF-*α* for 44 h were compared with those not incubated with TNF-α. As shown in Table 4, 7-deacetoxy-7-oxogedunin (**3**, IC_50_ = 7.3 µM), epoxyazadiradione (**8**, 10.2 µM), 17β-hydroxyazadiradione (**9**, 6.9 µM), and carapanolides C (**10**, 27.0 µM) and A (**17**, 25.3 µM) inhibited the decrease in cell viability with greater efficacy than silybin (IC_50_ = 37.2 µM) [36]. The structural requirements of gedunin-type limonoids for the activity were as follows; (i) compounds with a 7-keto group exhibited higher activity than those with 7α-acetoxy or 7α-hydroxy groups [7-deacetoxy-7-oxogedunin (**3**) > gedunin (**1**), 7-deacetoxygedunin (**4**)]; compounds with an α,β-epoxy or α,β-unsaturated cyclopropane moiety in the D-ring exhibited higher activity than those with an α,β-epoxy-δ-lactone moiety [epoxyazadiradione (**8**), 17β-hydroxyazadiradione (**9**) > **1**]. These structural requirements showed opposite tendencies to those related to NO production inhibitory activity, which is mentioned above.

## 3. Materials and Methods

### 3.1. General Experimental Procedures

The following instructions were used to obtain spectroscopic data: melting points, Yanagimoto micromelting point apparatus (Yanaco New Science Inc., Kyoto, Japan); optical rotations, JASCO DIP-1000 digital polarimeter (JASCO Co., Tokyo, Japan); IR spectra, PerkineElmer 1720X FTIR spectrophotometer (PerkineElmer Inc., Waltham, MA, USA); UV spectra, HITACHI U-2000 spectrometer (Hitachi High-Technologies Co., Tokyo, Japan) (acetonitrile as a solvent); ^1^H and ^13^C NMR spectra, Agilent VNMRS 600 spectrometer (Agilent Technologies Inc., Santa Clara, CA, USA) (CDCl_3_ was used as the solvent and TMS as the internal standard); FABMS, JEOL JMS-7000 mass spectrometer (JEOL Ltd., Tokyo, Japan); CD spectra, JASCO J-820 spectrometer (JASCO Co.); and HPLC, JASCO PU-1586 (RI 1531) (JASCO Co.). The following experimental conditions were used for column chromatography: (silica gel, 70–230 mesh; Merck, Darmstadt, Germany); medium-pressure liquid chromatography (MPLC; silica gel, 230–400 mesh; Merck); and TLC (silica gel 60 F_254_; Merck).

### 3.2. Material

The flower and seed oils of *C. guianensis* Aublet (Meliaceae), were collected in Amazon, Brazil in March of 2006, 2011, and 2013. Voucher specimens (CG-01-1, CGS-01-1, and CGS-01-2) were deposited at the Herbarium of the Laboratory of Medicinal Chemistry at Osaka University of Pharmaceutical Sciences as described previously [14,15,16,17,18,19,20,21,22,23].

### 3.3. Isolation of Compounds **1**–**3** from the Seed Oil of C. Guianensis

Preliminary silica gel column chromatography was performed to separate the seed oil (2.03 kg, CGS-01-2) of *C. guianensis* into eight fractions (Fractions A–H) [22]. Fraction C (29.3 g) was rechromatographed on a silica gel (70–230 mesh, 1.0 kg) column using *n*-hexane–EtOAc (5:1) to yield residues C3 (1.66 g) and C4 (1.02 g). Residue C3 (1.66 g) was rechromatographed on a silica gel (230–400 mesh, 100 g) column using *n*-hexane–EtOAc (3:1) to give a crystalline solid (590 mg), which was purified by HPLC [ODS, MeOH–H_2_O (70:30)] to afford compounds **1** (325 mg) and **2** (178 mg). Residue C4 (1.02 g) was rechromatographed on a silica gel (230–400 mesh, 50 g) column using *n*-hexane–EtOAc (3:1) to give a crystalline solid, which was recrystallized from MeOH to give compound **3** (510 mg). These isolates (**1**–**3**) were unambiguously identified by comparison of their physical and spectral data with those of authentic samples [14,15].

### 3.4. Reagents

LPS from *Salmonella enteritidis*, minimum essential medium (MEM), and William’s E medium were purchased from Sigma-Aldrich Chemical (St. Louis, MO, USA); fetal bovine serum (FBS) was from Life Technologies (Rockville, MD, USA); and other chemicals were from Wako Pure Chemical Industries, Co., Ltd. (Osaka, Japan). Microplates (96-well) were purchased from Sumitomo Bakelite Co., Ltd. (Tokyo, Japan).

### 3.5. Animals

Male ddY mice (Kiwa Laboratory Animal Co., Ltd., Wakayama, Japan) were housed at a constant temperature of 23 ± 2°C and were fed a standard laboratory chow (MF, Oriental Yeast Co., Ltd., Tokyo, Japan). The animals were fasted for 24 h prior to the beginning of the experiment but were allowed free access to tap water. All experiments were performed with conscious mice unless otherwise noted. The experimental protocol was approved by the Experimental Animal Research Committee of Kindai University (KAPR-26-001, 1 April 2014).

### 3.6. Effects on d-GalN/LPS-induced Liver Injury in Mice

The method described by Tiegs *et al*. [57] was modified and used for this study [33,35,36]. Curcumin [36] and silybin were used as reference compounds.

### 3.7. Effects on Cytotoxicity Induced by d-GalN in Primary Cultured Mouse Hepatocytes

Assay for the d-GalN-induced cytotoxicity in primary cultured mouse hepatocytes was performed as described previously [33,35,36]. Curcumin [26,27,29] and silybin [33,35,36] were used as reference compounds.

### 3.8. Effects on Production of NO in LPS-induced Mouse Peritoneal Macrophages

Assay for NO production in TGC-induced mouse peritoneal macrophages was performed as described previously [33,35,36]. *N*^G^-Monomethyl-L-arginine (L-NMMA) and caffeic acid phenethyl ester (CAPE) were used as reference compounds [33,36].

### 3.9. Effects on Cytotoxicity Induced by TNF-α in L929 Cells

Assay for the TNF-α-induced cytotoxicity in L929 cells was performed as described previously [33,35,36]. Silybin was used as a reference compound [36].

### 3.10. Statistics

All data are expressed as means ± S.E.M. The data analysis was performed with an one-way analysis of variance (ANOVA), followed by Dunnett’s test. Probability (*p*) values less than 0.05 were considered significant.

## 4. Conclusions

Three gedunin-type limonoids obtained from the flower oil of *C. guianensis*, gedunin (**1**), 6*α*-acetoxygedunin (**2**), and 7-deacetoxy-7-oxogedunin (**3**), showed protective effects against liver injury induced by d-GalN/LPS in mice at a dose of 25 mg/kg, p.o. The mechanisms of action are likely dependent on inhibition of LPS-induced macrophage activation and reduced sensitivity of hepatocytes to TNF-α; however, these compounds did not decrease the cytotoxicity caused by d-GalN. LPS-induced NO production is accompanied with production of several cytokines, such as TNF-α, IL-1, and IL-6, from macrophages through the toll-like receptor 4 (TLR4)-mediated pathways [58,59]. Recently, it was reported that **1** suppressed the activation of macrophages through binding to myeloid differentiation protein 2 (MD-2), and not by affecting TLR4-mediated signaling. Their data supports our results of inhibitory effects of **1** on NO production in LPS-activated macrophages [60]. In addition, the structural requirements of limonoids (**1**–**17**) with regard to inhibition of LPS-induced NO production in mouse peritoneal macrophages and TNF-α-induced cytotoxicity in L929 cells were found to show different tendencies as mentioned above. The detailed mechanisms of action for the hepatoprotective effects of limonoids need to be studied further.

## Figures and Tables

**Table 1:** Inhibitory effects of gedunin (**1**), 6α-acetoxygedunin (**2**), and 7-deacetoxy-7-oxogedunin (**3**) on d-GalN/LPS-induced liver injury in mice.

Treatment	Dose (mg/kg, p.o.)	*N*	sAST		sALT		Mortality
			(Karmen Unit)	Inhibition (%)	(Karmen Unit)	Inhibition (%)	
Normal (vehicle)	–	8	107 ± 9 **	–	20 ± 2 **	–	0/8
Control	–	12	5237 ± 1,000	–	8533 ± 1795	–	4/16
Gedunin (**1**)	25	7	2304 ± 651 *	56.2	2950 ± 710 *	65.7	0/7
	50	7	1923 ± 576 *	63.5	2824 ± 754 *	67.2	0/7
6α-Acetoxygedunin (**2**)	25	7	2384 ± 579 *	54.7	3120 ± 830 *	63.7	0/7
	50	7	1696 ± 580 **	67.9	2397 ± 873 **	72.2	0/7
7-Deacetoxy-7-oxo-	25	7	2093 ± 742 *	60.3	2899 ± 1024 *	66.3	0/7
gedunin (**3**)	50	6	1759 ± 579 *	66.7	2572 ± 903 **	70.2	1/7
Control	–	10	6033 ± 1647	–	6605 ± 1985	–	6/16
Curcumin [36]	12.5	10	4770 ± 1218	21.1	5024 ± 1189	24.0	0/10
	25	10	3177 ± 979	47.8	3253 ± 981	50.9	0/10
	50	9	2220 ± 563 *	63.8	1916 ± 483 *	71.2	1/10
Control	–	10	4709 ± 461	–	7088 ± 917	–	4/14
Silybin	500	8	1361 ± 191 **	71.1	1990 ± 439 **	71.9	0/8

Each value represents the mean ± S.E.M.; asterisks denote significant differences from the control group, * *p* < 0.05, ** *p* < 0.01.; commercial silybin was purchased from Funakoshi Co., Ltd. (Tokyo, Japan).

**Table 2:** Inhibitory effects of limonoids (**1**–**17**) on d-GalN-induced cytotoxicity in mouse primary hepatocytes.

Treatment	Inhibition (%)				
	0 µM	3 µM	10 µM	30 µM	100 µM
Gedunin (**1**)	0.0 ± 1.8	−8.4 ± 1.9	−3.9 ± 0.4	−3.2 ± 0.9	−5.2 ±0.2
6α-Acetoxygedunin (**2**)	0.0 ± 2.4	−2.6 ± 1.0	−1.9 ± 0.3	−2.5 ± 0.7	−1.8 ± 0.6
7-Deacetoxy-7-oxogedunin (**3**)	0.0 ± 2.2	−4.6 ± 0.5	−8.2 ± 0.9	−8.3 ± 1.1	−8.9 ± 0.6
7-Deacetoxy-7α-hydroxygedunin (**4**)	0.0 ± 1.6	−2.0 ± 1.4	−6.3 ± 0.5	−8.0 ± 0.4	−4.1 ± 0.7
Andirolide H (**5**)	0.0 ± 2.5	−6.2 ± 2.6	−9.0 ± 0.5	−9.2 ± 0.8	−0.6 ± 0.8
6α-Hydroxygedunin (**6**)	0.0 ± 2.0	−7.2 ± 2.4	−9.4 ± 0.8	−0.1 ± 0.7	−2.8 ± 0.5
Methyl angolensate (**7**)	0.0 ± 2.2	−2.1 ± 1.2	−7.5 ± 0.9	−6.3 ± 0.7	−6.6 ± 1.0
Epoxyazadiradione (**8**)	0.0 ± 2.1	−3.1 ± 8.9	−2.2 ± 2.5	−10.8 ± 0.6	−11.9 ± 0.3
17β-Hydroxyazadiradione (**9**)	0.0 ± 2.0	15.3 ± 2.3	−4.1 ± 1.2	−7.5 ± 1.4	−7.5 ± 4.0
Carapanolide C (**10**)	0.0 ± 1.4	8.0 ± 4.3	3.4 ± 4.2	6.7 ± 2.1	−7.7 ± 4.4
Carapanolide R (**11**)	0.0 ± 2.1	21.5 ± 2.8 **	27.8 ± 5.0 **	46.0 ± 4.7 **	36.0 ± 3.2 **
Carapanolide S (**12**)	0.0 ± 2.1	−7.8 ± 3.2	−3.8 ± 4.1	−3.7 ± 3.1	−7.1 ± 3.2
Carapanolide M (**13**)	0.0 ± 1.6	−7.0 ± 0.5	−7.3 ± 0.7	1.0 ± 0.4	−9.9 ± 1.0
Carapanolide Q (**14**)	0.0 ± 1.6	2.7 ± 1.9	−3.5 ± 2.9	−2.5 ± 2.1	−6.2 ± 1.7
Carapanolide O (**15**)	0.0 ± 1.9	7.5 ± 3.9	−5.3 ± 5.6	−5.2 ± 3.9	−2.1 ± 1.7
Guianolide A (**16**)	0.0 ± 3.7	9.2 ± 4.2	11.0 ± 5.3	9.8 ± 2.8	23.5 ± 3.5 **
Carapanolide A (**17**)	0.0 ± 2.0	−6.8 ± 1.2	−8.3 ± 0.7	−4.5 ± 0.6	−7.0 ± 0.6
Curcumin [26,27,29]	0.0 ± 3.7	0.1 ± 3.8	1.1 ± 2.2	−17.7 ± 1.3	−44.3 ± 0.3
Silybin [33,35,36]	0.0 ± 0.3	4.8 ± 1.1	7.7 ± 0.7	45.2 ± 8.8 **	77.0 ± 5.5 **

Each value represents the mean ± S.E.M. (*n* = 4); asterisks denote significant differences from the control group, ** *p* < 0.01.; commercial silybin was purchased from Funakoshi Co., Ltd. (Tokyo, Japan).

**Table 3:** Inhibitory effects of limonoids (**1**–**17**) on LPS-activated NO production in mouse peritoneal macrophages.

Treatment	Inhibition (%)					IC_50_
	0 µM	3 µM	10 µM	30 µM	100 µM	(µM)
Gedunin (**1**) [17]	0.0 ± 5.6 (100.0 ± 4.1)	25.1 ± 2.5 ** (102.2 ± 5.3)	84.5 ± 2.3 ** (119.5 ± 5.3)	101.8 ± 0.6 ** (94.8 ± 1.4)	100.9 ± 0.4 ** (3.0 ± 0.4 ^#^)	4.6
6*α*-Acetoxygedunin (**2**) [17]	0.0 ± 1.5 (100.0 ± 1.6)	16.9 ± 1.7 ** (96.8 ± 1.2)	67.6 ± 4.6 ** (102.3 ± 2.2)	88.4 ± 3.5 ** (92.5 ± 1.7)	99.6 ± 0.2 ** (53.6 ± 5.1 ^#^)	7.9
7-Deacetoxy-7-oxogedunin (**3**) [17]	0.0 ± 6.5 (100.0 ± 5.1)	7.4 ± 5.2 (100.3 ± 3.9)	40.9 ± 4.7 ** (98.9 ± 3.2)	94.0 ± 0.8 ** (98.8 ± 7.4)	88.1 ± 2.1 ** (83.7 ± 1.2)	12.8
7-Deacetoxy-7α-hydroxy-gedunin (**4**) [17]	0.0 ± 2.4 (100.0 ± 4.4)	15.7 ± 4.6 ** (110.3 ± 5.9)	55.7 ± 4.0 ** (106.6 ± 3.1)	98.8 ± 0.4 ** (96.3 ± 4.6)	100.2 ± 0.2 ** (2.6 ± 0.5 ^#^)	8.7
Andirolide H (**5**)	0.0 ± 5.6 (100.0 ± 1.8)	5.8 ± 6.1 (99.8 ± 4.5)	63.9 ± 3.0 ** (103.9 ± 6.9)	97.2 ± 0.9 ** (108.9 ± 2.4)	99.7 ± 0.5 ** (4.9 ± 0.5 ^#^)	9.4
6α-Hydroxygedunin (**6**) [17]	0.0 ± 6.2 (100.0 ± 4.5)	7.7 ± 7.1 (88.4 ± 3.0)	20.7 ± 4.3 ** (87.6 ± 4.0)	64.0 ± 3.1 ** (90.4 ± 2.6)	97.3 ± 0.3 ** (82.2 ± 4.2)	19.1
Methyl angolensate (**7**) [17]	0.0 ± 5.9 (100.0 ± 2.4)	10.1 ± 4.2 (108.8 ± 11.0)	20.0 ± 8.1 (108.8 ± 5.5)	42.2 ± 3.5 ** (111.0 ± 4.5)	24.0 ± 4.2 * (78.1 ± 5.3 ^#^)	> 100
Epoxyazadiradione (**8**)	0.0 ± 0.8 (100.0 ± 4.1)	10.5 ± 0.9 * (99.6 ± 2.9)	56.0 ± 4.0 ** (94.8 ± 2.3)	102.6 ±4.0 ** (81.9 ± 2.7)	112.3 ± 0.7 ** (10.0 ± 0.5 ^#^)	8.2
17β-Hydroxyazadiradione (**9**)	0.0 ± 4.9 (100.0 ± 1.8)	−10.4 ± 6.8 (95.4 ± 5.2)	9.4 ± 7.1 (94.4 ± 1.4)	65.1 ± 4.5 ** (94.8 ± 4.9)	97.4 ± 0.7 ** (84.8 ± 3.6)	20.3
Carapanolide C (**10**)	0.0 ± 2.6 (100.0 ± 3.4)	4.2 ± 8.8 (96.0 ± 4.1)	20.8 ± 5.0 ** (98.9 ± 3.4)	20.2 ± 4.9 (91.8 ± 2.8)	13.2 ± 1.9 (80.0 ± 4.4)	>100
Carapanolide R (**11**)	0.0 ± 1.3 (100.0 ± 1.0)	4.0 ± 2.2 (95.1 ± 1.8)	8.9 ± 2.3 (98.0 ± 1.7)	17.4 ± 1.3 (106.0 ± 1.9)	75.6 ± 1.2 ** (118.4 ± 1.0)	68.3
Carapanolide S (**12**)	0.0 ± 2.8 (100.0 ± 0.7)	2.5 ± 1.2 (97.9 ± 2.7)	15.9 ± 1.3 ** (93.8 ± 2.0)	72.2 ± 3.6 ** (96.8 ± 4.4)	99.8 ± 0.4 ** (73.7 ± 3.8 ^#^)	15.5
Carapanolide M (**13**)	0.0 ± 2.2 (100.0 ± 2.3)	−1.1 ± 2.7 (99.2 ± 0.6)	3.2 ± 2.8 (95.1 ± 1.3)	30.3 ± 3.1 ** (94.2 ± 2.9)	85.1 ± 1.5 ** (111.9 ± 1.6)	41.6
Carapanolide Q (**14**)	0.0 ± 2.4 (100.0 ± 2.8)	1.9 ± 0.7 (99.8 ± 1.6)	14.4 ± 2.2 (96.3 ± 1.6)	44.3 ± 1.1 ** (95.5 ± 4.1)	75.3 ± 3.5 ** (115.5 ± 2.2)	38.0
Carapanolide O (**15**)	0.0 ± 2.5 (100.0 ± 4.6)	−2.5 ± 5.4 (104.1 ± 2.5)	14.7 ± 8.2 (107.9 ± 2.7)	6.9 ± 5.0 (106.7 ± 2.5)	102.5 ± 3.2 ** (108.7 ± 5.2)	46.0
Guianolide A (**16**)	0.0 ± 1.8 (100.9 ± 0.9)	1.9 ± 3.2 (96.9 ± 1.8)	3.2 ± 1.4 (101.5 ± 1.9)	12.7 ± 1.6 (98.7 ± 1.8)	71.3 ± 3.2 ** (106.2 ± 1.6)	77.9
Carapanolide A (**17**)	0.0 ± 1.5 (100.0 ± 1.8)	−0.6 ± 2.1 (103.0 ± 2.6)	1.2 ± 2.0 (103.9 ± 4.3)	41.1 ± 1.0 ** (109.5 ± 4.3)	4.9 ± 1.8 (91.3 ± 1.6)	> 100
l-NMMA [33,36]	0.0 ± 3.1 (100.0 ± 0.9)	1.4 ± 2.8 (101.1 ± 5.7)	19.9 ± 2.8 ** (100.7 ± 6.2)	43.0 ± 2.1 ** (102.6 ± 4.2)	70.9 ± 1.6 ** (106.4 ± 4.6)	36.0
CAPE [33,36]	0.0 ± 2.1 (100.0 ± 1.5)	5.9 ± 5.2 (95.4 ± 0.7)	44.4 ± 3.2 ** (70.0 ± 4.0 ^#^)	86.2 ± 1.1 ** (71.4 ± 6.0 ^#^)	99.6 ± 0.1 ** (53.0 ± 1.4 ^#^)	11.0

Each value represents the mean ± S.E.M. (*n* = 4); asterisks denote significant differences from the control group, * *p* < 0.05, ** *p* < 0.01; ^#^ cytotoxic effects were observed, and values in parentheses indicate cell viability (%) in MTT assay; commercial silybin was purchased from Funakoshi Co., Ltd. (Tokyo, Japan), whereas L-NMMA and CAPE were from Sigma-Aldrich Chemical Co., LLC. (St. Louis, MO, USA).

**Table 4:** Inhibitory effects of limonoids (**1**–**17**) on TNF-*α*-induced cytotoxicity in L929 cells.

Treatment	Inhibition (%)				
	0 µM	1 µM	3 µM	10 µM	30 µM
Gedunin (**1**)	0.0 ± 2.1	4.5 ± 1.9	21.8 ± 3.7 **	38.1 ± 3.8 **	36.5 ± 4.1 **
6α-Acetoxygedunin (**2**)	0.0 ± 1.6	10.9 ± 1.0 **	23.2 ± 1.8 **	36.3 ± 2.1 **	37.3 ± 1.4 **
7-Deacetoxy-7-oxogedunin (**3**)	0.0 ± 1.1	5.8 ± 1.5	26.7 ± 4.5 **	58.6 ± 7.2 **	68.7 ± 4.8 **
7-Deacetoxy-7α-hydroxygedunin (**4**)	0.0 ± 0.3		−6.5 ± 2.4	2.7 ± 2.3	36.5 ± 1.8 **
Andirolide H (**5**)	0.0 ± 0.8	−6.6 ± 3.6	−0.7 ± 1.2	7.6 ± 1.1	39.2 ± 1.7 **
6α-Hydroxygedunin (**6**)	0.0 ± 1.3	8.1 ± 1.9	6.7 ± 1.5	12.1 ± 3.0	28.3 ± 1.7 **
Methyl angolensate (**7**)	0.0 ± 1.4	−0.5 ± 3.5	0.6 ± 2.9	13.3 ± 2.6 *	24.6 ± 2.9 **
Epoxyazadiradione (**8**)	0.0 ± 5.3		13.7 ± 3.9	39.1 ± 6.5 **	91.5 ± 11.4 **
17β-Hydroxyazadiradione (**9**)	0.0 ± 1.5	14.1 ± 3.4	23.9 ± 3.9 **	64.0 ± 3.3 **	91.3 ± 8.2 **
Carapanolide C (**10**)	0.0 ± 3.7	4.9 ± 2.1	14.2 ± 3.2	27.7 ± 4.3 **	54.5 ± 5.5 **
Carapanolide R (**11**)	0.0 ± 4.1		−6.3 ± 4.7	−1.3 ± 3.8	31.7 ± 3.8 **
Carapanolide S (**12**)	0.0 ± 1.5		−5.5 ± 2.2	−1.4 ± 1.5	−2.5 ± 1.2
Carapanolide M (**13**)	0.0 ± 6.5		−1.5 ± 7.1	7.0 ± 4.4	−5.1 ± 6.2
Carapanolide Q (**14**)	0.0 ± 5.5		8.6 ± 4.4	1.3 ± 4.2	9.2 ± 2.5
Carapanolide O (**15**)	0.0 ± 6.5		6.3 ± 4.3	1.0 ± 6.4	1.5 ± 4.1
Guianolide A (**16**)	0.0 ± 2.9		−6.2 ± 5.2	−4.5 ± 1.9	−7.3 ± 3.0
Carapanolide A (**17**)	0.0 ± 3.7		8.8 ± 6.5	21.5 ± 5.5 **	58.2 ± 4.7 **
**Treatment**	**Inhibition (%)**				
	0 µM	3 µM	10 µM	30 µM	100 µM
Silybin [36]	0.0 ± 2.6	5.3 ± 2.8	22.0 ± 3.8 **	48.0 ± 4.1 **	50.8 ± 3.9 **

Each value represents the mean ± S.E.M. (*n* = 4); asterisks denote significant differences from the control group, * *p* < 0.05, ** *p* < 0.01.; commercial silybin was purchased from Funakoshi Co., Ltd. (Tokyo, Japan).
